# Social media use and academic performance among college students: the chain mediating roles of social anxiety and fear of missing out and the moderating effect of teacher-student relationship

**DOI:** 10.3389/fpsyg.2025.1649890

**Published:** 2025-10-14

**Authors:** Zhuliu Gong, Yi Guo, Jinghao Tan

**Affiliations:** School of Journalism and Communication, Chongqing University, Chongqing, China

**Keywords:** college students, social media use, academic performance, fear of missing out, social anxiety, teacher-student relationships

## Abstract

**Objective:**

To explore the mechanisms by which social media use affects academic performance among college students, examining the chain mediation effects of social anxiety and fear of missing out (FoMO), as well as the moderating role of teacher-student relationships.

**Methods:**

Using stratified cluster sampling, questionnaire data were collected from 3,716 Chinese undergraduate students aged 18–24. The questionnaires measured social media usage frequency, social anxiety, FoMO, teacher-student relationships, and academic performance. Scale reliability and validity were first examined through confirmatory factor analysis, followed by structural equation modeling to assess chain mediation effects. The moderating effect of teacher-student relationships was tested using the PROCESS Model 14 framework.

**Results:**

Social media use had a significant negative direct effect on college students’ academic performance. Social anxiety and FoMO served as significant mediators in this relationship. Teacher-student relationships significantly moderated the negative effect of FoMO on academic performance.

**Conclusion:**

High-frequency social media use among college students undermines academic performance through an emotional pathway whereby social anxiety leads to increased FoMO. Strong teacher-student relationships can function as an emotional regulatory mechanism, significantly reducing this adverse impact.

## 1 Introduction

With the rapid development of media technologies, it has become increasingly common for adolescents to form “disembedded” connections with the world through social media platforms. According to the 55th Statistical Report on China’s Internet Development by the China Internet Network Information Center (CNNIC), the number of internet users in China has reached 1.108 billion, with an internet penetration rate of 78.6%. Among these users, 1.101 billion individuals engage with social networks, comprising 99.3% of the total internet user population. Notably, adolescents aged 10–29 make up 26.1% of the total user base, underscoring the prominence of young people in the digital landscape (China Internet Network Information Center, 2025). This statistic aligns with Prensky’s (2001) concept of “digital natives,” highlighting the heavy reliance of young people on social media in both daily and academic life.

*The China Youth Big Data Insight Report* (2022) reveals that young people aged 18–24 spend an average of 5.8 h online daily, illustrating the habitual nature of social media use and its deep emotional integration into their lives (China Mobile Think Tank and Wutong Big Data, 2022). A recent survey demonstrated that Chinese university students spend over 5 h per day on mobile phones, with approximately four-fifths (79%) using smartphones during class time (Li et al., 2020). This pervasive use of social media has significantly impacted the educational sphere, where platforms now serve as essential tools for communication between teachers and students, for accessing academic resources, and for sharing research interests (Zhao, 2023). However, whether social media enhances or hinders learning remains a topic of ongoing debate in academic circles.

The body of research on the effects of social media can be categorized into three interconnected theoretical perspectives. First, the debate between technological optimism and pessimism. Advocates of technological optimism highlight how decentralized platforms empower users by challenging traditional educational hierarchies and broadening access to learning resources. In contrast, critics argue that algorithmic surveillance and data control can lead to an identity crisis and other negative outcomes (Liang and Chen, 2025). Second, the issue of technological dependence and alienation. The constant connectivity provided by social media fragments attention and blurs individual values, with excessive use linked to changes in neuroplasticity and a diminished perception of reality (Lau, 2017). Third, the relationship between media use and mental health. While the drive for technological adaptation is often praised, it can trigger “digital anxiety” and disrupt academic and life outcomes through problematic internet use (Wu, 1998).

When it comes to academic performance (Al-Yafi et al., 2018), the findings of existing research are often contradictory. On one hand, social media is viewed as a tool that can enhance learning by providing decentralized access to knowledge and fostering informal learning communities. It also serves as a valuable resource for expanding academic information channels (Chen et al., 2021; Owusu, 2020). On the other hand, excessive immersion in virtual social interactions can deplete cognitive resources, leading to reduced time and energy for academic engagement (Evers et al., 2020). Social media multitasking has been shown to have a direct negative effect on academic performance (Lau, 2017). This paradoxical relationship necessitates further investigation, particularly through the lens of psychological mechanisms that may mediate these effects.

## 2 Literature review and hypotheses

### 2.1 Social media use and academic performance among college students

While empirical research demonstrates that social media use positively correlates with academic performance when deeply integrated into instructional processes as a medium for knowledge construction and teacher-student interaction (Altalhi, 2020), additional evidence supports this relationship. Alshalawi’s (2022) quantitative study of Saudi undergraduate students further confirmed a positive association between social media usage frequency and academic performance, highlighting its potential value as a digital learning tool. However, the effects of social media use are context-dependent. When students multitask between social media and academic activities, cognitive resource conflicts produce negative outcomes (Malik et al., 2020; Shafiq and Parveen, 2023). Substantial research links high-intensity social media use with poor academic outcomes (Whelan et al., 2020; Evers et al., 2020) and shows that excessive use can impair attention and increase academic distraction (Boer et al., 2020). Experimental studies demonstrate that social media multitasking directly reduces test performance by increasing cognitive load (Lau, 2017). Meta-analytic evidence confirms these detrimental effects are robust across contexts (Appel et al., 2020). Based on this evidence, we hypothesize:

*H1:* Social media use negatively affects college students’ academic performance.

### 2.2 Social media use, social anxiety, and college students’ academic performance

Social anxiety shows complex relationships with digital media use. The American Psychiatric Association defines this condition through two key dimensions: persistent fear or anxiety in social situations and excessive worry about negative evaluation by others (Lolk, 2013). Clinical research characterizes social anxiety as an anxiety disorder marked by anticipatory embarrassment and shame (Rasouli et al., 2022).

Problematic social media use disrupts both learning efficiency and real-world relationships while worsening anxiety symptoms by weakening offline social support (Alkis et al., 2017). Digital natives are particularly vulnerable to compulsive online behaviors that trigger emotional disorders including social anxiety and depression (Salmela-Aro et al., 2017). Social anxiety undermines university students’ psychosocial adaptation and relationship quality while predicting poor academic performance (Brook and Willoughby, 2015; Zukerman et al., 2019; Mou et al., 2024). Socially anxious individuals often use self-isolation and avoidance to manage their anxiety (Russell and Topham, 2012). These avoidance behaviors—withdrawing from classroom participation, avoiding questions, and reluctance to engage in discussions—produce maladaptive outcomes and absenteeism that ultimately harm academic performance (Mou et al., 2024). We therefore propose:

*H2:* Social anxiety mediates the relationship between social media use and college students’ academic performance.

### 2.3 Social media use, fear of missing out, and college students’ academic performance

Fear of missing out (FoMO) occurs when individuals worry about missing relevant information or desired content when social media and smartphones are inaccessible (Casale et al., 2018). Research shows that problematic social media behaviors significantly influence adolescents’ FoMO levels. Studies find strong links between problematic social media use and FoMO, with more severe usage patterns associated with greater FoMO symptoms (Primack et al., 2017).

Student-focused research indicates that FoMO negatively affects academic performances (Tanrikulu and Mouratidis, 2023; Parizad et al., 2022; Kong et al., 2024). This relationship operates through specific cognitive mechanisms: when students lack effective strategies for managing classroom smartphone use and experience impaired decision-making, their learning focus deteriorates and academic performance suffers (Rosen et al., 2018). We therefore hypothesize:

*H3:* Fear of missing out (FoMO) mediates the relationship between social media use and college students’ academic performance.

### 2.4 Social media use, fear of missing out, social anxiety, and college students’ academic performance

Despite promises to “give people the power to build community and bring the world closer together,” social media often disrupts daily activities and relationships. Social media use links to negative mental health outcomes (Vannucci et al., 2017), with FoMO and social anxiety as key psychological mechanisms (Scheinfeld and Voorhees, 2022; Elhai et al., 2016).

FoMO amplifies uncertainty and anxiety in virtual social contexts (Oliveira et al., 2018; Zhu et al., 2024). These negative emotions drive pathological online compensation behaviors—frequent social media refreshing and excessive message checking—creating compulsive dependence on virtual social activities (Wang et al., 2021). This creates a vicious cycle where cognitive resources and emotional energy are depleted, leading to higher online social anxiety levels (Wu et al., 2025).

Research consistently shows that higher social anxiety predicts lower academic performance over time (Kajastus et al., 2024; Ashshawareb et al., 2024; Abdulsalim et al., 2025). Socially anxious students struggle to integrate into academic environments because they fear not being valued in interpersonal relationships. These fears inhibit classroom participation and help-seeking from teaching assistants, professors, and other college staff (Brook and Willoughby, 2015). Therefore, we propose:

*H4:* FoMO and social anxiety sequentially mediate the relationship between social media use and college students’ academic performance.

### 2.5 The moderating role of teacher-student relationships

University environments systematically influence young adults’ cognitive development, psychological adjustment, and social adaptation (Thornberg and Charmaz, 2022). Teacher-student relationships form core interpersonal bonds in educational settings, establishing psychological connections through cognitive interaction, emotional bonding, and behavioral feedback (Wang et al., 2023). Attachment theory shows that early interactions with significant others create internalized working models that guide behavioral responses across contexts (Wang et al., 2023).

Research demonstrates that teacher-student relationship quality significantly affects students’ classroom engagement, academic performance, and intrinsic motivation (Pianta et al., 2012). Positive relationships enhance learning engagement and performance through emotional support and behavioral modeling while buffering against negative emotions like anxiety and depression. Teacher behaviors shape students’ development in cognitive, emotional, and behavioral domains through protective factors including mutual respect, trust, and emotional warmth (Veldman et al., 2013). Poor teacher-student relationships weaken learning motivation and academic self-efficacy, creating sustained declines in learning outcomes (Wang et al., 2024). We hypothesize:

*H5:* Teacher-student relationships moderate the relationship between social media use and college students’ academic performance.

## 3 Methods

### 3.1 Survey method

We used stratified cluster sampling to ensure representative national coverage. From March 15 to April 1, 2025, we selected 120 cities across China using random sampling principles. The selection included provincial capitals across 23 provinces and 5 autonomous regions, as well as four municipalities directly under central government administration. Additionally, 2–6 prefecture-level cities were randomly selected from non-capital administrative regions within each province and autonomous region. To achieve optimal demographic distribution, quota sampling was implemented for participant recruitment. Research assistants were responsible for questionnaire distribution and collection among designated target populations, resulting in 3,775 distributed questionnaires. To ensure data quality, incomplete responses, surveys with excessively short completion times, and those containing extreme or anomalous values were systematically excluded. Following rigorous data cleaning procedures, 3,716 valid responses were retained, yielding an effective response rate of 98.47%.

The research assistants (RAs) recruited for this study were required to have at least a second-year undergraduate education in relevant fields such as psychology, communication, or education. RAs were selected based on their familiarity with the survey process, communication skills, and ability to effectively manage participant interactions. Prior to data collection, all RAs underwent a comprehensive training program that covered ethical research practices, appropriate survey management techniques, and quality control protocols. Data collection involved recruiting at least one RA or survey team in each sample city. Individual RAs were responsible for collecting 30 to 90 responses, while survey teams managed between 100 and 200 questionnaires. The survey was administered through the online platform Wenjuanxing^1^ via one-on-one, face-to-face interactions. Participants accessed the surveys through the provided links, with RAs ensuring informed consent was obtained and assigning unique identification numbers to each response. In cases where participants had cognitive capacity but limited mobility, RAs conducted verbal, one-on-one interviews and recorded the responses on their behalf. Following data collection, two independent reviewers performed logical checks and data screening to ensure the accuracy and integrity of the data.

The final sample comprised 1,842 males (49.6%) and 1,874 females (50.4%), with 1,939 urban residents (52.2%) and 1,777 rural residents (47.8%). Age distribution included 655 eighteen-year-olds (17.6%), 746 nineteen-year-olds (20.0%), 751 twenty-year-olds (20.2%), 732 twenty-one-year-olds (19.8%), 739 twenty-two-year-olds (19.9%), and 93 participants over twenty-two years (2.5%), as detailed in Table 1.

**Table 1:** Sample demographic characteristics.

Variable	Category	Frequency	Percentage (%)
Gender	Male	1,842	49.6
	Female	1,874	50.4
Residence	Urban	1,939	52.2
	Non-urban	1,777	47.8
Age	18 years	655	17.6
	19 years	746	20.0
	20 years	751	20.2
	21 years	732	19.8
	22 years	739	19.9
	>22 years	93	2.5

Inclusion criteria: (1) aged 18–24 years; (2) Chinese nationality; (3) permanent Chinese residents (annual time abroad ≤1 month); (4) voluntary participation with informed consent; (5) ability to complete online surveys independently or with research assistant support; (6) comprehension of questionnaire item meanings.

Exclusion criteria: (1) impaired consciousness or psychiatric disorders; (2) concurrent participation in similar research studies; (3) unwillingness to cooperate.

### 3.2 Questionnaire design and variable measurement

The research team conducted comprehensive literature review to identify existing questionnaire designs and clarify research questions and relevant variables based on study objectives. Initial questionnaire drafts were developed with systematic item organization and categorization. Pilot testing was subsequently conducted to examine questionnaire validity and item appropriateness, with iterative refinements based on testing results culminating in the final instrument. The distributed questionnaire comprised two primary sections: demographic variables to assess participants’ basic characteristics, including gender, current educational level, household registration type, and age, specifically tailored for adolescent populations; and measurement scales for study variables addressing research-specific content.

According to the study’s theoretical framework, five primary variables were examined: social media use, teacher-student relationships, social anxiety, fear of missing out, and academic performance. To ensure adequate reliability and validity, items were primarily adapted from previously validated and well-established scales. Additionally, demographic questionnaires were included to examine variations across gender and geographic regions.

#### 3.2.1 Social media use scale

Social media usage was assessed using the Social Media Use Integration Scale (SMUIS) developed by Jenkins-Guarnieri et al. (2013). The scale comprises two dimensions: Social Integration and Emotional Connection (6 items) and Integration into Social Routines (4 items), utilizing a 5-point Likert scale (1 = strongly disagree, 5 = strongly agree). Higher total scores indicate greater social media dependence and emotional investment. Confirmatory factor analysis revealed excellent internal consistency (*α* = 0.893), with sub-dimension Cronbach’s *α* values of 0.819 for Social Integration and Emotional Connection and 0.857 for Integration into Social Routines, demonstrating superior scale reliability.

#### 3.2.2 Teacher-student relationship scale

Teacher-student relationships were measured using the standardized instrument revised by Zhang et al. (2012), originally based on Pianta’s (2001) Student-Teacher Relationship Scale and culturally adapted by Guo et al. (2017). The revised version encompasses four dimensions: teacher-student satisfaction, closeness, supportiveness, and conflict, comprising 18 items with 5-point Likert scoring. Conflict dimension items employed reverse scoring (1 = completely inconsistent, 5 = completely consistent), while remaining dimensions utilized positive scoring. Internal consistency for the total scale was *α* = 0.802, with sub-dimension *α* values ranging from 0.802 to 0.865, indicating cross-cultural applicability and robust construct validity.

#### 3.2.3 Social anxiety scale

Social anxiety assessment utilized Leary’s (1983) Interaction Anxiousness Scale (IAS), which focuses on subjective tension experiences in spontaneous social situations. The 15-item scale employs 5-point scoring (1 = completely inconsistent to 5 = completely consistent), designed according to two principles: emphasis on emotional experiences rather than overt behaviors, and situational settings involving unpredictable spontaneous interactions. The Cronbach’s *α* coefficient for the current sample was 0.818, indicating excellent scale reliability.

#### 3.2.4 FoMO scale

FoMO was measured using the standardized scale developed by Przybylski et al. (2013), comprising 10 items with 5-point scoring (1 = strongly disagree to 5 = strongly agree). The instrument assesses individuals’ concerns about missing important online information or social activities, with total scores positively correlated with FoMO levels. Internal consistency for the FoMO scale was *α* = 0.836, meeting reliability requirements.

#### 3.2.5 Academic performance scale

Academic performance was assessed using self-report measures rather than objective GPA data due to privacy restrictions and institutional policies. Specifically, academic performance was evaluated through a five-item scale adapted from Cunningham (2021). These items were measured on a five-point Likert scale, ranging from strongly disagree (1) to strongly agree (5). The items were appropriately modified to fit the context of the present study, ensuring relevance to the surveyed population of Chinese university students.

The internal consistency of the scale was assessed using Cronbach’s *α* coefficient, which yielded a value of 0.845, indicating a high level of reliability within the current sample. While the use of self-reported measures is common in academic research (Hornstra et al., 2023; Rožman et al., 2025) it is essential to acknowledge the potential limitations associated with this approach. Self-reports may be influenced by social desirability biases or self-perception errors, which can introduce inaccuracies into the measurement of academic performance. To address these concerns, future studies could benefit from integrating objective performance indicators, such as grade point average (GPA) or instructor evaluations, alongside self-reported data, thereby improving the overall validity of the findings.

## 4 Results

### 4.1 Common method bias testing

This study employed Harman’s single-factor test to systematically examine potential common method bias. Statistical analysis revealed that without factor rotation, six common factors with eigenvalues greater than 1 were extracted, with the first factor accounting for 32.364% of the variance. This value falls substantially below the critical threshold of 40%, indicating that the research data does not exhibit significant systematic common method bias and that the measurements demonstrate satisfactory discriminant validity. These findings provide methodological validity assurance for subsequent structural equation modeling analyses and ensure the reliability of inferences regarding inter-variable relationships.

### 4.2 Confirmatory factor analysis

This study utilized AMOS 26.0 statistical software to conduct confirmatory factor analysis on five core latent variables, with model fit indices detailed in Table 2. The analysis demonstrated that the five-factor model achieved a chi-square to degrees of freedom ratio (*χ*^2^/df) of 2.473 (below the critical value of 3), a root mean square error of approximation (RMSEA) of 0.034 (below the threshold of 0.08), and comparative fit index (CFI), Tucker-Lewis index (TLI), and goodness of fit index (GFI) values all exceeding 0.90. These fit indices not only fully satisfy statistical requirements but also demonstrate superior fit compared to alternative competing models, indicating robust discriminant validity in this study.

**Table 2:** Confirmatory factor analysis.

Model	*χ*^2^/df	RMSEA	CFI	TLI	GFI
Five-factor model	2.473	0.034	0.956	0.943	0.975
Four-factor model	3.439	0.078	0.901	0.883	0.889
Three-factor model	4.264	0.198	0.769	0.752	0.79
Two-factor model	4.815	0.327	0.713	0.695	0.699
Single-factor model	5.391	0.339	0.66	0.642	0.659

### 4.3 Descriptive statistics and correlation analysis

The means, standard deviations, and correlation coefficients for all variables are presented in Table 3. Results indicated that social media usage intensity demonstrated a significant positive correlation with fear of missing out (*r* = 0.172, *p* < 0.05), as well as significant positive associations with social anxiety (*r* = 0.165, *p* < 0.01) and teacher-student relationship quality (*r* = 0.156, *p* < 0.01), while exhibiting a significant negative correlation with academic performance (*r* = −0.131, *p* < 0.01). Regarding mediating variables, fear of missing out showed a moderate positive correlation with social anxiety (*r* = 0.173, *p* < 0.01), a significant negative association with academic performance (*r* = −0.361, *p* < 0.01), and a significant negative correlation with teacher-student relationship quality (*r* = −0.138, *p* < 0.05). Social anxiety demonstrated negative associations with both academic performance (*r* = −0.249, *p* < 0.01) and teacher-student relationship quality (*r* = −0.256, *p* < 0.01), while academic performance exhibited a significant positive correlation with teacher-student relationship quality (*r* = 0.234, *p* < 0.01). These correlation patterns preliminarily reveal complex pathways among variables, providing empirical foundations for subsequent structural equation modeling.

**Table 3:** Descriptive statistics and correlation analysis.

Variable	M	SD	Social media use	FoMO	Social anxiety	Academic performance	Teacher-student relationship
Social media use	3.105	0.954	1				
FoMO	2.874	0.577	0.172*	1			
Social anxiety	2.353	0.725	0.165**	0.173**	1		
Academic performance	2.146	0.987	−0.131**	−0.361**	−0.249**	1	
Teacher-student relationship	2.249	0.662	0.156**	−0.138*	−0.256**	0.234**	1

*p < 0.05, **p < 0.01.

### 4.4 Main effects and mediation analysis

**Figure 1:**
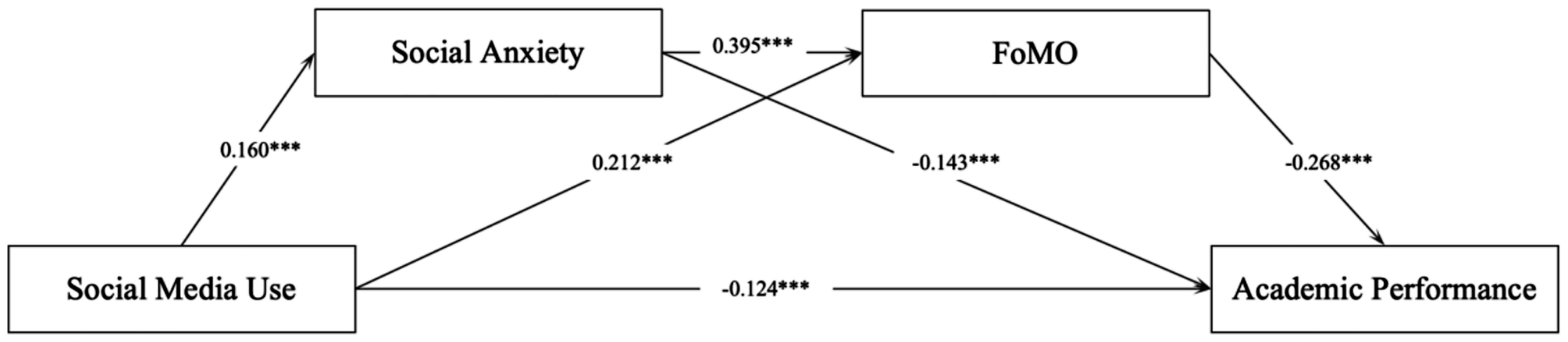
Structural equation model path coefficients.

Based on the conceptual model, this study constructed a structural equation model incorporating social media usage, social anxiety, fear of missing out, and academic performance as core variables. In testing the hypothesized path relationships, social media usage demonstrated a significant positive predictive effect on fear of missing out (*β* = 0.212, *p* < 0.001) and social anxiety (*β* = 0.160, *p* < 0.001), while exhibiting a significant negative predictive relationship with academic performance (*β* = −0.124, *p* < 0.001), thereby supporting the first research hypothesis. Further analysis revealed that social anxiety exerted a significant positive effect on fear of missing out (*β* = 0.395, *p* < 0.001) and a significant negative predictive effect on academic performance (*β* = −0.143, *p* < 0.001), while fear of missing out similarly demonstrated a significant negative predictive relationship with academic performance (*β* = −0.268, *p* < 0.001). All hypothesized paths in the model achieved statistical significance, with path directions consistent with research expectations. Path coefficients and significance levels are detailed in Table 4 and Figure 1.

**Table 4:** Model path relationship hypothesis testing results.

Path relationship	Estimate	S.E.	C.R.	*p*-value	Conclusion
Social media use → FoMO	0.212	0.022	12.824	<0.001	Support
Social media use → social anxiety	0.160	0.018	7.566	<0.001	Support
Social media use → academic performance	−0.124	0.015	−6.326	<0.001	Support
Social anxiety → FoMO	0.395	0.017	20.405	<0.001	Support
Social anxiety → academic performance	−0.143	0.012	−5.792	<0.001	Support
FoMO → academic performance	−0.268	0.017	−16.187	<0.001	Support

Based on the research’s conceptual model, this study hypothesized that social anxiety and fear of missing out would mediate the relationship between adolescent social media usage and academic performance, with analysis and verification conducted using AMOS 28.0. The bias-corrected nonparametric percentile bootstrap method was employed to examine the mediation effects of social anxiety and fear of missing out. Table 5 presents standardized estimates for each indirect path and 95% confidence intervals for mediation effects, with mediation effects considered significant when 95% confidence intervals exclude zero. According to Table 5, the 95% confidence intervals for all three indirect paths exclude zero, indicating significant effects for social anxiety mediation, fear of missing out mediation, and serial mediation. Specifically, the mediation effect value for social anxiety was 0.0075, for fear of missing out was 0.0165, and for serial mediation was 0.0041. Therefore, Hypotheses 2, 3, and 4 are all supported.

**Table 5:** Mediation path relationship testing results.

Path	Standardized path coefficient	Standard error	95% Confidence Interval	
			Lower	Bound
Social media use → social anxiety → academic performance	0.0075	0.0033	0.0034	0.0125
Social media use → FoMO → academic performance	0.0165	0.0058	0.0069	0.0164
Social media use → social anxiety → FoMO → Academic performance	0.0041	0.0016	0.0021	0.0074

### 4.5 Moderation analysis

**Figure 2:**
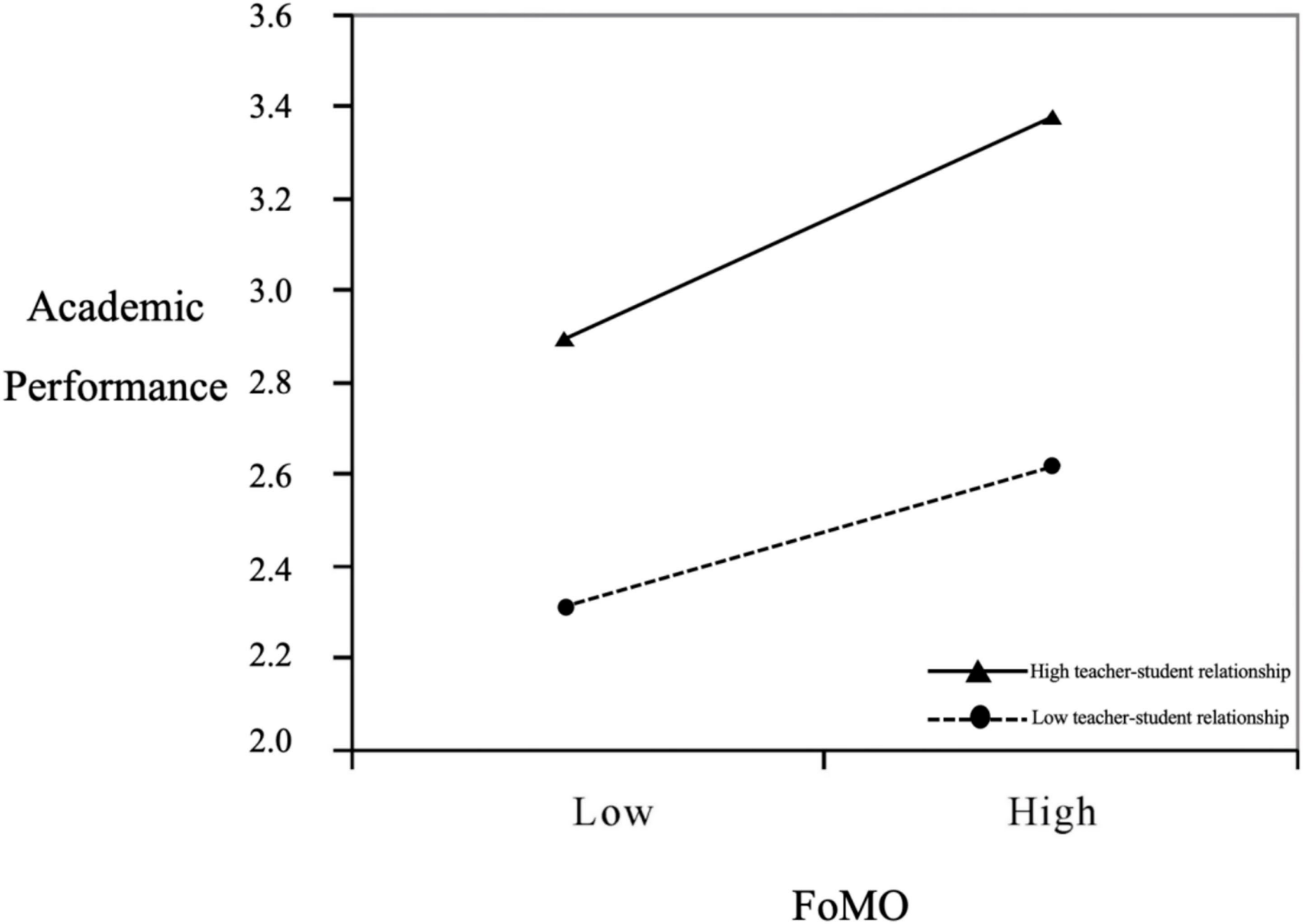
Moderating effects of teacher-student relationship quality on the association between fear of missing out and academic performance.

The PROCESS macro was utilized to examine the moderating role of teacher-student relationships in the association between fear of missing out and academic performance. Results indicated that the interaction term between teacher-student relationships and fear of missing out exhibited a significant negative predictive effect on academic performance (*β* = −0.175, *p* < 0.01), demonstrating that teacher-student relationships negatively moderate the relationship between fear of missing out and academic performance. Therefore, Hypothesis H5 was supported. To provide a more intuitive representation of the moderating effect of teacher-student relationships on the fear of missing out-academic performance relationship, this study followed recommendations by Aiken et al. (1991) by plotting relationships at one standard deviation above and below the mean to illustrate differential effects of fear of missing out on academic performance across varying levels of teacher-student relationship quality, as shown in Figure 2.

## 5 Discussion

### 5.1 Direct effects of problematic internet use on academic performance

The current study demonstrates that social media use significantly and negatively impacts college students’ academic performance. Higher social media usage is associated with a reduced likelihood of achieving favorable academic outcomes, which aligns with findings from previous studies (Evers et al., 2020; Sampasa-Kanyinga et al., 2019; Giunchiglia et al., 2018). This negative relationship can be examined through various theoretical lenses. From a behavioral media use perspective, social media platforms are mobile and operate in real-time, allowing students to access them almost anywhere, including during class. This “always-on” nature of social media can undermine sustained attention and the cognitive processing necessary for effective learning (Al-Menayes, 2014).

From the media psychology perspective, constant information streams create patterns of “dynamic updating, immediate response, and constant switching,” leading to fragmented attention and cognitive overload (Shen, 2019). As a result, students’ working memory is occupied, making it difficult for them to focus deeply on course material. Furthermore, frequent late-night use of social media is closely linked to sleep disorders and academic burnout (Evers et al., 2020). According to the emotional exhaustion model, chronic sleep deprivation and fatigue drain students’ motivation and emotional regulation, ultimately leading to decreased academic engagement and poorer grades.

### 5.2 Mediating effects of social anxiety and fear of missing out

The study also finds that social anxiety and FoMO play significant mediating roles in the relationship between social media use and academic performance. According to cognitive-behavioral theory, individuals with social anxiety tend to have negative automatic thoughts about social interactions (Clark and Wells, 1995; Weidman and Levinson, 2015), making them more likely to turn to social media as a way to avoid real-world social pressures (Lee and Stapinski, 2012). However, this form of social participation does not alleviate social anxiety. Instead, it consumes cognitive resources, weakening students’ attention and executive control during learning tasks (Junco and Cotten, 2012; Oberst et al., 2017; Albulescu et al., 2024). Additionally, socially anxious individuals often engage in upward social comparisons on social media, leading to increased feelings of inferiority and negative self-worth, which further detracts from academic motivation and performance (Kajastus et al., 2024; Vogel et al., 2014; Jehi et al., 2024).

Further research indicates that excessive social media use intensifies anxiety and stress levels among college students, diminishing the positive impact of self-control on academic performance (Vogel et al., 2014). Thus, social anxiety emerges as a crucial mediating variable, influencing both the emotional and cognitive aspects of students’ academic performance.

FoMO, which is significantly linked to social media use (Alutaybi et al., 2020), exacerbates this problem. Students with high FoMO levels tend to worry about missing out on their peers’ experiences, which disrupts their focus and academic engagement (Chai et al., 2018). This, in turn, leads to poor academic performance and negative student outcomes (Hairul et al., 2025). This study is the first to validate this mediating pathway within the Chinese context.

Theoretical frameworks like Self-Determination Theory (SDT) and Social Comparison Theory shed light on the mechanisms underlying these relationships. SDT suggests that social media environments often fail to satisfy students’ needs for autonomy, competence, and relatedness, prompting emotional compensation through excessive media use. This, in turn, diverts time from academic activities (Ryan and Deci, 2000; Deci and Ryan, 2008). Social Comparison Theory explains that upward comparisons on social media reinforce negative self-perceptions, amplifying FoMO’s detrimental effects on academic motivation (Vogel et al., 2014). Together, social anxiety and FoMO exert a negative influence on academic performance by depleting cognitive resources and reducing motivation.

### 5.3 Chain mediating effects of social anxiety and fear of missing out

The chain mediation analysis reveals that social media use influences academic performance through a series of mediating factors: social media use leads to social anxiety, which then leads to FoMO, and ultimately harms academic outcomes. This finding supports previous research (Dempsey et al., 2019; Wolniewicz et al., 2018) and extends the “emotional activation—cognitive resource displacement” pathway outlined in the I-PACE model (Brand et al., 2016).

Studies have shown that social anxiety directly predicts FoMO, with individuals exhibiting higher levels of social anxiety also showing a stronger concern about missing out on social media content (Dempsey et al., 2019). From a media psychology perspective, social anxiety can be seen as an immediate emotional response to perceived evaluative threats. Students with higher levels of social anxiety often monitor peer feedback closely and avoid negative evaluations, which intensifies their fear of missing out on relevant social information (Dempsey et al., 2019). FoMO then becomes a motivational amplifier, driving students to frequently refresh their social media feeds and switch attention between tasks, which compromises their ability to focus on academic work. While this model offers valuable insights, it may not fully capture the complexity of the emotional and cognitive processes involved, as it assumes a linear progression that may not apply universally across all individuals.

### 5.4 Moderating effects of teacher-student relationships

Positive teacher-student relationships (TSR) can be a crucial factor in satisfying students’ “relatedness needs,” as emphasized by Self-Determination Theory. When students feel respected, understood, and supported by their teachers, their intrinsic motivation is strengthened, and their sense of control over academic goals increases. This reduces the compulsive use of social media driven by FoMO and improves executive function and time management skills, reducing attention fragmentation. Empirical evidence shows that in the presence of strong TSR, the negative impact of FoMO on academic performance is significantly reduced (Li et al., 2022; Hornstra et al., 2023).

Positive TSR can be understood through the lens of James Carey’s ritual view of communication (Carey, 1975), where teacher-student interactions are seen as continuous, meaningful, and ritualistic rather than sporadic exchanges of information. Unlike the fragmented interactions common in online spaces, in-person interactions, such as classroom discussions, after-class tutoring, and daily greetings, help satisfy students’ relatedness needs, providing them with emotional security. This stability can counteract the cognitive depletion caused by FoMO, allowing students to better focus on their academic tasks. According to Feng (2022), the moderating effects of TSR unfold in three ways: emotional support, cognitive reappraisal, and resource reallocation.

First, emotional support helps students feel secure and valued, which can reduce the emotional triggers of FoMO. Second, cognitive reappraisal helps students challenge their fears of missing out on information, reducing their anxiety. Third, resource reallocation helps students better allocate their attention and time, thus reducing distractions caused by FoMO and enhancing their ability to engage in focused learning.

In practical terms, while improving TSR may help alleviate some of the cognitive and emotional burdens caused by FoMO, it does not fully counteract the negative effects on academic performance. To put this into perspective, this modest effect can be compared to the role of a supportive friend who helps to mitigate stress but does not eliminate it entirely. In educational settings, this implies that while fostering positive teacher-student relationships can certainly play a role in managing the adverse effects of FoMO, it might need to be combined with other interventions, such as developing self-regulation skills or digital detox practices, to more effectively address the issue. Educational and psychological interventions aimed at strengthening teacher-student relationships should focus on providing emotional support and fostering trust and understanding between students and teachers. However, the modest nature of this effect indicates that these interventions should not be seen as a panacea for all the challenges posed by FoMO. A more holistic approach is needed, where TSR interventions are complemented by strategies that directly address time management, media literacy, and emotional regulation.

## 6 Conclusion

While existing research suggests that social media use may impair university students’ academic achievement, the underlying mechanisms remain insufficiently understood. This study employed social media use as the independent variable, positioning social anxiety and fear of missing out as sequential mediators, and incorporating teacher-student relationships as a moderating factor within the Self-Determination Theory framework. This approach enabled the construction of a multilayered model encompassing usage behaviors, emotional processes, and academic outcomes. The theoretical foundation of this study integrates three complementary frameworks operating at distinct analytical levels. Self-Determination Theory (SDT) provides the foundational motivational framework, explaining how social media environments may undermine students’ psychological needs for autonomy, competence, and relatedness. Social Comparison Theory operates at the cognitive processing level, elucidating how upward social comparisons trigger negative self-evaluations and academic motivation deficits. The Interaction of Person-Affect-Cognition-Execution (I-PACE) model functions at the behavioral execution level, describing how emotional and cognitive disruptions translate into maladaptive usage patterns that interfere with academic engagement.

The findings reveal that higher frequencies of social media use are associated with elevated levels of social anxiety among students, which subsequently triggers stronger fear of missing out, ultimately producing a suppressive effect on academic performance. Concurrently, the results demonstrate that more positive teacher-student relationships attenuate the negative impact of fear of missing out on academic outcomes. These conclusions underscore the critical importance of enhancing teacher-student emotional connections and disrupting digital emotional chains to preserve academic performance.

## 7 Limitations

Several methodological limitations should be acknowledged when interpreting these findings. The cross-sectional design precludes definitive causal inferences, as reverse causation remains possible—students with academic difficulties may increase social media use as avoidance, or those with pre-existing anxiety may be more drawn to problematic online behaviors. Reliance on self-reported measures across all variables introduces potential biases, including social desirability bias and inaccurate self-perception for academic performance assessment, as well as recall bias for social media usage frequency. Future studies should incorporate objective measures such as actual GPA records and digital usage tracking data to enhance measurement validity.

Additionally, generalizability may be limited to Chinese university students aged 18–24, as cultural factors and educational system characteristics specific to the Chinese context may influence the observed relationships. The study did not account for potentially important confounding variables including academic major, socioeconomic status, prior academic achievement, personality traits, sleep quality, and pre-existing mental health conditions, which could moderate or mediate the examined relationships and may have led to overestimation of effects. Finally, while teacher-student relationships moderated the FoMO-academic performance relationship, the modest effect size suggests this protective factor alone may be insufficient to counteract negative impacts of problematic social media use, warranting investigation of additional protective factors in future research.

## Data Availability

The raw data supporting the conclusions of this article will be made available by the authors without undue reservation.

## Footnotes

^1^ https://www.wjx.cn

## References

[ref1] Abdulsalim S. Anaam M. S. Farooqui M. Alshammari M. S. Alfadly S. Alolayan J.. (2025). Evaluation of social media addiction and its relationship with anxiety and academic performance among medical and non-medical students: a cross-sectional study from Saudi Arabia. Healthcare 13:295. doi: 10.3390/healthcare13030295, PMID: 39942484 PMC11817380

[ref2] Aiken L. S. West S. G. Reno R. R. (1991). Multiple regression: testing and interpreting interactions, Newbury Park, CA: Sage.

[ref3] Albulescu I. Labar A. V. Manea A. D. Stan C. (2024). The mediating role of cognitive test anxiety on the relationship between academic procrastination and subjective wellbeing and academic performance. Front. Public Health 12:1336002. doi: 10.3389/fpubh.2024.1336002, PMID: 38919925 PMC11196964

[ref4] Alkis Y. Kadirhan Z. Sat M. (2017). Development and validation of social anxiety scale for social media users. Comput. Human Behav. 72, 296–303. doi: 10.1016/j.chb.2017.03.011

[ref5] Al-Menayes J. (2014). The relationship between mobile social media use and academic performance in university students. New Media Mass Commun. 25, 23–29.

[ref6] Alshalawi A. S. (2022). Social media usage intensity and academic performance among undergraduate students in Saudi Arabia. Contemp. Educ. Technol. 14:ep361. doi: 10.30935/cedtech/11711

[ref7] Altalhi N. (2020). SNS as an educational tool: effect on academic performance and learners’ perceptions (Doctoral dissertation): Nova Southeastern University.

[ref8] Alutaybi A. Al-Thani D. McAlaney J. Ali R. (2020). Combating fear of missing out (FoMO) on social media: the FoMO-R method. Int. J. Environ. Res. Public Health 17:6128. doi: 10.3390/ijerph17176128 32842553 PMC7504117

[ref9] Al-Yafi K. El-Masri M. Tsai R. (2018). The effects of using social network sites on academic performance: the case of Qatar. J. Enterp. Inf. Manag. 31, 446–462. doi: 10.1108/JEIM-08-2017-0118

[ref10] Appel M. Marker C. Gnambs T. (2020). Are social media ruining our lives? A review of meta-analytic evidence. Rev. Gen. Psychol. 24, 60–74. doi: 10.1177/1089268019880891

[ref12] Ashshawareb I. J. Mo’en S. A. ALi D. A. Mustafa N. A. Ashshawareb Y. I. (2024). Psychological anxiety of COVID-19 pandemic and its effect on academic performance of university students. J. Ecohuman. 3, 1169–1180. doi: 10.62754/joe.v3i6.4083

[ref13] Boer M. Stevens G. Finkenauer C. Eijnden R. (2020). Attention deficit hyperactivity disorder-symptoms, social media use intensity, and social media use problems in adolescents: investigating directionality. Child Dev. 91, e853–e865. doi: 10.1111/cdev.13334, PMID: 31654398 PMC7497191

[ref14] Brand M. Young K. S. Laier C. Wölfling K. Potenza M. N. (2016). Integrating psychological and neurobiological considerations regarding the development and maintenance of specific internet-use disorders: an interaction of person-affect-cognition-execution (I-PACE) model. Neurosci. Biobehav. Rev. 71, 252–266. doi: 10.1016/j.neubiorev.2016.08.033, PMID: 27590829

[ref15] Brook C. A. Willoughby T. (2015). The social ties that bind: social anxiety and academic achievement across the university years. J. Youth Adolesc. 44, 1139–1152. doi: 10.1007/s10964-015-0262-8, PMID: 25691148

[ref16] Carey J. W. (1975). A cultural approach to communication. Theorizing communication. Readings across traditions, Thousand Oaks: Sage Publications, 37–50.

[ref17] Casale S. Rugai L. Fioravanti G. (2018). Exploring the role of positive metacognitions in explaining the association between the fear of missing out and social media addiction. Addict. Behav. 85, 83–87. doi: 10.1016/j.addbeh.2018.05.020, PMID: 29864680

[ref18] Chai H. Y. Niu G. F. Chu X. W. Wei Q. Song Y. H. Sun X. J. (2018). Fear of missing out: what have I missed again? Adv. Psychol. Sci. 26, 527–537. doi: 10.3724/sp.j.1042.2018.00527

[ref19] Chen X. Ma J. Wei J. Yang S. (2021). The role of perceived integration in WeChat usages for seeking information and sharing comments: a social capital perspective. Inf. Manag. 58:103280. doi: 10.1016/j.im.2020.103280

[ref20] China Internet Network Information Center. (2025). China internet network information center released the 55th <statistical report on China’s internet development> in Beijing. Available online at: https://cnnic.cn/n4/2025/0117/c208-11228.html (Accessed May 24, 2025).

[ref21] China Mobile Think Tank and Wutong Big Data. (2022). The China youth big data insight report. Available online at: https://www.sohu.com/a/615217302_483389 (Accessed May 24, 2025).

[ref22] Clark D. M. Wells A. (1995). “A cognitive model of social phobia” in Social phobia: diagnosis, assessment, and treatment. eds. Heimberg R. G. Liebowitz M. R. Hope D. A. Schneier F. R. (New York: Guilford Press), 69–93.

[ref23] Cunningham M. (2021). An investigation into the relationship between perceived academic performance, depression, anxiety, and stress; gender differences (Doctoral dissertation). Dublin: National College of Ireland.

[ref25] Deci E. L. Ryan R. M. (2008). Self-determination theory: a macrotheory of human motivation, development, and health. Can. Psychol. 49, 182–185. doi: 10.1037/a0012801

[ref26] Dempsey A. E. O’Brien K. D. Tiamiyu M. F. Elhai J. D. (2019). Fear of missing out (FoMO) and rumination mediate relations between social anxiety and problematic Facebook use. Addict. Behav. Rep. 9:100150. doi: 10.1016/j.abrep.2018.100150 31193746 PMC6542373

[ref27] Elhai J. D. Levine J. C. Dvorak R. D. Hall B. J. (2016). Fear of missing out, need for touch, anxiety and depression are related to problematic smartphone use. Comput. Human Behav. 63, 509–516. doi: 10.1016/j.chb.2016.05.079

[ref28] Evers K. Chen S. Rothmann S. Dhir A. Pallesen S. (2020). Investigating the relation among disturbed sleep due to social media use, school burnout, and academic performance. J. Adolesc. 84, 156–164. doi: 10.1016/j.adolescence.2020.08.011, PMID: 32920331

[ref29] Feng M. Y. (2022). How "communication" meets "ritual": an interpretation of James W. Carey’s communication thought from a religious perspective. J. Journal. Commun. Res. 29, 19–37. Available online at: https://kns.cnki.net/kcms2/article/abstract?v=Ss1McYY34CeSoK4bWgQmahZ4sFCJa9cxFiBR6H4cploru6Of9SDbFTQmzttAcREdZky9OMy43qpUa6SeuXgdAMlKztiwHctu-XhM3IAOu8gsEUH5

[ref30] Giunchiglia F. Zeni M. Gobbi E. Bignotti E. Bison I. (2018). Mobile social media usage and academic performance. Comput. Human Behav. 82, 177–185. doi: 10.1016/j.chb.2017.12.041

[ref9006] Guo M. J. Liu R. D. Zhen R.. (2017). The effect of parent-child attachment on subjective well-being in middle school students: The chain mediating roles of teacher-student relationship and self-esteem. Studies of Psychology and Behavior, 15, 351–358. Available online at: https://kns.cnki.net/kcms2/article/abstract?v=Ss1McYY34Cf3TsA9hqr80RilGLEZ8_WoT7xnKc9y9acTJ_IlzvfWCCcDiLV7bNdnbrtd0erW7_mcnQNpDDzpYpUag6NNHgbacOdcDEh0g6I3aPH85VIW4_37sdWIU9HtDs68xVw8N0MNMZxekTKfsIx7GOxz4q-vV0Qh8SNqvEs=&uniplatform=NZKPT&language=CHS

[ref31] Hairul M. H. Hasbullah N. Q. Jamaludin N. S. Mohd Isa S. H. Zamsari Z. Hassan M. S.. (2025). Personal and social determinants of fear of missing out (FoMO) among university students in UiTM Lendu. e-J. Media Soc. (e-JOMS), 8, 82–100. Available online at: https://ir.uitm.edu.my/id/eprint/113812

[ref33] Hornstra L. Stroet K. Rubie-Davies C. Flint A. (2023). Teacher expectations and self-determination theory: considering convergence and divergence of theories. Educ. Psychol. Rev. 35:76. doi: 10.1007/s10648-023-09788-4

[ref34] Jehi T. Mulvey M. Shulgan E. Burke E. Dean M. Betancourt J.. (2024). Anxiety, depression, stress, and test anxiety are inversely associated with academic performance among undergraduate students post-COVID-19 confinement. Am. J. Health Educ. 55, 89–99. doi: 10.1080/19325037.2023.2296943

[ref9005] Jenkins-Guarnieri M. A. Wright S. L. Johnson B. (2013). Development and validation of a social media use integration scale. Psychology of popular media culture, 2:38.

[ref35] Junco R. Cotten S. R. (2012). No a 4 u: the relationship between multitasking and academic performance. Comput. Educ. 59, 505–514. doi: 10.1016/j.compedu.2011.12.023

[ref36] Kajastus K. Haravuori H. Kiviruusu O. Marttunen M. Ranta K. (2024). Associations of generalized anxiety and social anxiety with perceived difficulties in school in the adolescent general population. J. Adolesc. 96, 291–304. doi: 10.1002/jad.12275, PMID: 37985185

[ref37] Kong L. Sun H. He W. Hu W. (2024). Distraction or motivation? Unraveling the role of fear of missing out on college students’ learning engagement. BMC Psychol. 12, 1–13. doi: 10.1186/s40359-024-02164-z, PMID: 39578856 PMC11583681

[ref38] Lau W. W. F. (2017). Effects of social media usage and social media multitasking on the academic performance of university students. Comput. Human Behav. 68, 286–291. doi: 10.1016/j.chb.2016.11.043

[ref9004] Leary M. R. (1983). Social anxiousness: The construct and its measurement. Journal of Personality Assessment, 47, 66–75. 6834234 10.1207/s15327752jpa4701_8

[ref39] Lee B. W. Stapinski L. A. (2012). Seeking safety on the internet: relationship between social anxiety and problematic internet use. J. Anxiety Disord. 26, 197–205. doi: 10.1016/j.janxdis.2011.11.001, PMID: 22169053

[ref40] Li X. Bergin C. Olsen A. A. (2022). Positive teacher-student relationships may lead to better teaching. Learn. Instr. 80:101581. doi: 10.1016/j.learninstruc.2022.101581

[ref41] Li L. Lok G. K. I. Mei S. L. Cui X. L. Li L. Ng C. H.. (2020). The severity of mobile phone addiction and its relationship with quality of life in Chinese university students. PeerJ 8:e8859. doi: 10.7717/peerj.8859, PMID: 32547849 PMC7271884

[ref42] Liang W. Chen F. (2025). Circles of “intricacy”: the learning dilemmas of gen Z college students and their formation logic. China Youth Study 5, 21–28. doi: 10.19633/j.cnki.11-2579/d.2025.0045

[ref43] Lolk A. (2013). “Neurokognitive lidelser” in Diagnostic and statistical manual of mental disorders (Arlington, VA: American Psychiatric Association).

[ref44] Malik M. J. Ahmad M. Kamran M. R. Aliza K. Elahi M. Z. (2020). Student use of social media, academic performance, and creativity: the mediating role of intrinsic motivation. Interact. Technol. Smart Educ. 17, 403–415. doi: 10.1108/ITSE-01-2020-0005

[ref46] Mou Q. Zhuang J. Wu Q. Zhong Y. Dai Q. Cao X.. (2024). Social media addiction and academic engagement as serial mediators between social anxiety and academic performance among college students. BMC Psychol. 12:190. doi: 10.1186/s40359-024-01635-7, PMID: 38582933 PMC10998323

[ref48] Oberst U. Wegmann E. Stodt B. Brand M. Chamarro A. (2017). Negative consequences from heavy social networking in adolescents: the mediating role of fear of missing out. J. Adolesc. 55, 51–60. doi: 10.1016/j.adolescence.2016.12.008, PMID: 28033503

[ref49] Oliveira L. M. Bermudez M. B. de Amorim Macedo M. J. Passos I. C. (2018). Comorbid social anxiety disorder in patients with alcohol use disorder: a systematic review. J. Psychiatr. Res. 106, 8–14. doi: 10.1016/j.jpsychires.2018.09.008, PMID: 30236640

[ref50] Owusu P. K. (2020). Online platform for teaching and learning in improving student performance (a case study of Ghana technology university college). Middle East J. Appl. Sci. Technol. 3, 75–83. Available online at: https://mejast.com/data/uploads/90012.pdf

[ref51] Parizad N. Radfar M. Yarmohammadi M. Alinejad V. (2022). Smartphone addiction and its relationship with loneliness, fear of missing out, and academic performance among students of Urmia University of medical sciences. Nurs. Midwifery J. 20, 43–54. doi: 10.52547/unmf.20.1.43

[ref52] Pianta R. C. Hamre B. K. Allen J. P. (2012). “Teacherstudent relationships and engagement: conceptualizing, measuring, and improving the capacity of classroom interactions” in Handbook of research on student engagement. eds. Christenson S. L. Reschly A. L. Wylie C. (MA: Springer), 365–386.

[ref53] Prensky M. (2001). Digital natives, digital immigrants part 2: do they really think differently? On the Horizon 9, 1–6. doi: 10.1108/10748120110424843

[ref54] Primack B. A. Shensa A. Escobar-Viera C. G. Barrett E. L. Sidani J. E. Colditz J. B.. (2017). Use of multiple social media platforms and symptoms of depression and anxiety: a nationally-representative study among US young adults. Comput. Hum. Behav. 69, 1–9. doi: 10.1016/j.chb.2016.11.013 PMC13268370 42305949

[ref9003] Pianta R. C. (2001). Student-teacher relationship scale: Professional manual. Lutz, FL: Psychological Assessment Resources.

[ref9002] Przybylski A. K. Murayama K. DeHaan C. R. Gladwell V. (2013). Motivational, emotional, and behavioral correlates of fear of missing out. Computers in Human Behavior, 29, 1841–1848.

[ref55] Rasouli S. Gupta G. Nilsen E. Dautenhahn K. (2022). Potential applications of social robots in robot-assisted interventions for social anxiety. Int. J. Soc. Robot. 14, 1–32. doi: 10.1007/s12369-021-00851-0, PMID: 35096198 PMC8787185

[ref56] Rosen L. D. Carrier L. M. Pedroza J. A. Elias S. O’Brien K. M. Lozano J.. (2018). The role of executive functioning and technological anxiety (FOMO) in college course performance as mediated by technology usage and multitasking habits. Psicol. Educ. 24, 14–25. doi: 10.5093/psed2018a3 PMC8048369 33867798

[ref57] Rožman M. Vrečko I. Tominc P. (2025). Psychological factors impacting academic performance among business studies’ students. Educ. Sci. 15:121. doi: 10.3390/educsci15020121

[ref58] Russell G. Topham P. (2012). The impact of social anxiety on student learning and well-being in higher education. J. Ment. Health 21, 375–385. doi: 10.3109/09638237.2012.694505, PMID: 22823093

[ref59] Ryan R. M. Deci E. L. (2000). Self-determination theory and the facilitation of intrinsic motivation, social development, and well-being. Am. Psychol. 55, 68–78. doi: 10.1037/0003-066X.55.1.68, PMID: 11392867

[ref60] Salmela-Aro K. Upadyaya K. Hakkarainen K. Lonka K. Alho K. (2017). The dark side of internet use: two longitudinal studies of excessive internet use, depressive symptoms, school burnout and engagement among Finnish early and late adolescents. J. Youth Adolesc. 46, 343–357. doi: 10.1007/s10964-016-0494-2, PMID: 27138172

[ref61] Sampasa-Kanyinga H. Chaput J. P. Hamilton H. A. (2019). Social media use, school connectedness, and academic performance among adolescents. J. Prim. Prev. 40, 189–211. doi: 10.1007/s10935-019-00543-6 30796583

[ref62] Scheinfeld E. Voorhees H. L. (2022). How social media, FoMO, and isolation influence our perceptions of others who “break the rules”. Soc. Media Soc. 8:20563051221103841. doi: 10.1177/20563051221103841

[ref63] Shafiq M. Parveen K. (2023). Social media usage: analyzing its effect on academic performance and engagement of higher education students. Int. J. Educ. Dev. 98:102738. doi: 10.1016/j.ijedudev.2023.102738

[ref64] Shen J. (2019). Social-media use and academic performance among undergraduates in biology. Biochem. Mol. Biol. Educ. 47, 615–619. doi: 10.1002/bmb.21293, PMID: 31454138

[ref66] Tanrikulu G. Mouratidis A. (2023). Life aspirations, school engagement, social anxiety, social media use and fear of missing out among adolescents. Curr. Psychol. 42, 28689–28699. doi: 10.1007/s12144-022-03917-y

[ref67] Thornberg R. Charmaz K. (2022). “Designing grounded theory studies” in The SAGE handbook of qualitative research design. ed. Flick U. (Thousand Oaks, CA: Sage), 452–466.

[ref69] Vannucci A. Flannery K. M. Ohannessian C. M. (2017). Social media use and anxiety in emerging adults. J. Affect. Disord. 207, 163–166. doi: 10.1016/j.jad.2016.08.040 27723539

[ref70] Veldman I. vanTartwijk J. Brekelmans M. Wubbels T. (2013). Job satisfaction and teacher-student relationships across the teaching career: four case studies. Teach. Teach. Educ. 32, 55–65. doi: 10.1016/j.tate.2013.01.005

[ref71] Vogel E. A. Rose J. P. Roberts L. R. Eckles K. (2014). Social comparison, social media, and self-esteem. Psychol. Pop. Media Cult. 3, 206–222. doi: 10.1037/ppm0000047

[ref72] Wang P. Gan X. Li H. Jin X. (2023). Parental marital conflict and internet gaming disorder among Chinese adolescents: the multiple mediating roles of deviant peer affiliation and teacher-student relationship. PLoS One 18:e0280302. doi: 10.1371/journal.pone.0280302, PMID: 36649287 PMC9844898

[ref73] Wang J. S. Liu W. L. Li Q. (2021). Mobile phone addiction and sleep quality in college students: the roles of anxiety and ruminant thinking. Chin. J. Clin. Psych. 29, 1060–1063. doi: 10.16128/j.cnki.1005-3611.2021.05.034.

[ref74] Wang Y. Wang L. Yang L. Wang W. (2024). Influence of perceived social support and academic self-efficacy on teacher-student relationships and learning engagement for enhanced didactical outcomes. Sci. Rep. 14:28396. doi: 10.1038/s41598-024-78402-6, PMID: 39551776 PMC11570595

[ref75] Weidman A. C. Levinson C. A. (2015). I’m still socially anxious online: offline relationship impairment characterizing social anxiety manifests and is accurately perceived in online social networking profiles. Comput. Human Behav. 49, 12–19. doi: 10.1016/j.chb.2014.12.045

[ref76] Whelan E. Islam A. K. M. N. Brooks S. (2020). Applying the SOBC paradigm to explain how social media overload affects academic performance. Comput. Educ. 143:103692. doi: 10.1016/j.compedu.2019.103692

[ref77] Wolniewicz C. A. Tiamiyu M. F. Weeks J. W. Elhai J. D. (2018). Problematic smartphone use and relations with negative affect, fear of missing out, and fear of negative and positive evaluation. Psychiatry Res. 262, 618–623. doi: 10.1016/j.psychres.2017.09.058, PMID: 28982630

[ref78] Wu B. (1998). Lonely carnival: Communication in the digital age. Beijing, China: China Renmin University Press, 334–335. doi: 10.3390/bs15010084

[ref79] Wu W. Zhang J. Jo N. (2025). Fear of missing out and online social anxiety in university students: mediation by irrational procrastination and media multitasking. Behav. Sci. 15:84. 39851888 10.3390/bs15010084 PMC11762956

[ref9001] Zhang W. J. Cheng Y. J. Zou H. Yang Y. (2012). The impact of emotional intelligence and social problem-solving skills on teacher-student relationships among middle school students. Psychological Science, 35, 624–630. doi: 10.16719/j.cnki.1671-6981.2012.03.018

[ref80] Zhao L. (2023). Social media multitasking and college students' academic performance: a situation–organism–behavior–consequence perspective. Psychol. Sch. 60, 3151–3168. doi: 10.1002/pits.22912

[ref81] Zhu X. Lian W. Fan L. (2024). Network analysis of internet addiction, online social anxiety, fear of missing out, and interpersonal sensitivity among Chinese university students. Depress. Anxiety 2024, 1–14. doi: 10.1155/2024/5447802 PMC11918617 40226693

[ref82] Zukerman G. Yahav G. Ben-Itzchak E. (2019). Diametrically opposed associations between academic achievement and social anxiety among university students with and without autism spectrum disorder. Autism Res. 12, 1376–1385. doi: 10.1002/aur.2129, PMID: 31115185

